# Establishment and validation of a bad outcomes prediction model based on EEG and clinical parameters in prolonged disorder of consciousness

**DOI:** 10.3389/fnhum.2024.1387471

**Published:** 2024-06-17

**Authors:** Wanqing Liu, Yongkun Guo, Jingwei Xie, Yanzhi Wu, Dexiao Zhao, Zhe Xing, Xudong Fu, Shaolong Zhou, Hengwei Zhang, Xinjun Wang

**Affiliations:** ^1^Department of Neurosurgery, The Fifth Affiliated Hospital of Zhengzhou University, Zhengzhou, China; ^2^Henan Engineering Research Center for Prevention and Treatment of Brain Injuries, Zhengzhou, China; ^3^Henan Key Laboratory of Brain Science and Brain Computer Interface Technology, Zhengzhou, China; ^4^Department of Neurosurgery, The Third Affiliated Hospital of Zhengzhou University, Zhengzhou, China

**Keywords:** EEG, prognosis, clinical prediction, prolonged disorder of consciousness, nomogram

## Abstract

**Objective:**

This study aimed to explore the electroencephalogram (EEG) indicators and clinical factors that may lead to poor prognosis in patients with prolonged disorder of consciousness (pDOC), and establish and verify a clinical predictive model based on these factors.

**Methods:**

This study included 134 patients suffering from prolonged disorder of consciousness enrolled in our department of neurosurgery. We collected the data of sex, age, etiology, coma recovery scales (CRS-R) score, complications, blood routine, liver function, coagulation and other laboratory tests, resting EEG data and follow-up after discharge. These patients were divided into two groups: training set (*n* = 107) and verification set (*n* = 27). These patients were divided into a training set of 107 and a validation set of 27 for this study. Univariate and multivariate regression analysis were used to determine the factors affecting the poor prognosis of pDOC and to establish nomogram model. We use the receiver operating characteristic (ROC) and calibration curves to quantitatively test the effectiveness of the training set and the verification set. In order to further verify the clinical practical value of the model, we use decision curve analysis (DCA) to evaluate the model.

**Result:**

The results from univariate and multivariate logistic regression analyses suggested that an increased frequency of occurrence microstate A, reduced CRS-R scores at the time of admission, the presence of episodes associated with paroxysmal sympathetic hyperactivity (PSH), and decreased fibrinogen levels all function as independent prognostic factors. These factors were used to construct the nomogram. The training and verification sets had areas under the curve of 0.854 and 0.920, respectively. Calibration curves and DCA demonstrated good model performance and significant clinical benefits in both sets.

**Conclusion:**

This study is based on the use of clinically available and low-cost clinical indicators combined with EEG to construct a highly applicable and accurate model for predicting the adverse prognosis of patients with prolonged disorder of consciousness. It provides an objective and reliable tool for clinicians to evaluate the prognosis of prolonged disorder of consciousness, and helps clinicians to provide personalized clinical care and decision-making for patients with prolonged disorder of consciousness and their families.

## 1 Introduction

Disorders of consciousness (DOC) refer to conditions that change a person’s state of consciousness due to damage or malfunction in the sections of the nervous system that control arousal and consciousness. Prolonged disorder of consciousness (pDOC) are defined as a loss of consciousness for more than 28 days (Eapen et al., 2017). Coma is the most severe form of impaired consciousness. Those in a coma could possibly perish, fully regain awareness, or transition into a middle-ground state like unresponsive wakefulness syndrome/vegetative state (UWS/VS) or minimally conscious state (MCS) (Septien and Rubin, 2018). The treatment of pDOC requires a lot of medical resources, long-term hospitalization will not only bring huge economic costs to patients, but also bring great pressure to patients’ physical and mental health. In clinical work, we often face prognosis consultation from the families of pDOC patients. Accurate prognosis prediction is very important for patients and their families, and it is also of great significance for clinicians and nurses. The prognostic model can help clinicians to more effectively transform aggregate statistics into individual patient nursing decisions (Song et al., 2018). In this context, predicting the evolution of pDOC and determining reliable prognostic indicators is an important link for doctors to make clinical decisions.

As far as we know, there are some prognostic models for patients with pDOC. Song et al. (2018) used resting-state functional magnetic resonance imaging of the brain to predict 1 year outcomes in patients with pDOC. Yang et al. (2020) demonstrated that the structure of sleep electroencephalogram correlates with the short-term prognosis of patients with pDOC. Anderson et al. (2020) utilized three biomarkers, GFAP, UCH-L1, and MAP-2, to predict recovery at 6 months in patients with pDOC. Xiong et al. (2022) developed a prognostic model and found that higher serum albumin concentrations, higher levels of consciousness, younger age, and the presence of pupillary reflexes predicted improvement in pDOC at 6 months after injury. A 3 year outcome prediction column chart for poor prognosis of DOC has been constructed by some researchers using four predictor variables (age, GCS score, state of consciousness, and BAEP grade) and one outcome variable (the Glasgow Outcome Scale (GOS)) (Kang et al., 2022). While many studies have identified several neurophysiologic or neuroimaging risk factors in patients with pDOC, the aforementioned tests are difficult to perform, relatively expensive, and time-consuming. Their use is not widespread due to these limitations. And electroencephalogram (EEG) is one of the few available mobile techniques available, and therefore EEG is more amenable to bedside monitoring or diagnosis in patients with pDOC.

The dynamic pattern of the EEG can be called a “microstate,” which refers to the scalp potential topography or the global pattern of the scalp potential topology. This pattern will change over time according to certain rules and is orderly and dynamic on the whole, which is of great significance for understanding the rules of brain activity (Wang et al., 2023). Because the EEG microstate pattern has a high temporal resolution, it has great advantages in measuring rapid dynamic changes in the brain, thereby more accurately detecting the temporal dynamic evolution of the whole-brain neuronal network (Tarailis et al., 2024). Gui et al. (2020) used microstates to assess language processing in pDOC patients and combined them with a language stimulation paradigm to predict the recovery of pDOC patients with good predictive classification. In addition, the EEG microstate is a suitable candidate biomarker, and its time scale is faster than power spectrum or functional network analysis (Tait et al., 2020). Single objective EEG marker will make doctors rely too much on EEG and ignore clinical features, and then affect the judgment of the prognosis of patients. At the same time, the latest international guidelines for the diagnosis of pDOC patients recommend the use of both clinical and instrumental assessments to minimize the risk of misdiagnosis (Giacino et al., 2018; Kondziella et al., 2020). In this case, it is necessary to combine EEG with clinical factors to improve the accuracy of evaluating the prognosis of patients with pDOC.

Therefore, from a clinical point of view, this study combines the microstate of EEG with clinical general data, behavioral scores and laboratory tests to predict the poor prognosis of patients with pDOC and based on clinical factors (that is, clinical indicators, serological markers, electrophysiological indicators) to establish a practical, comprehensive and accurate clinical prediction model, so that the poor prognosis of patients with pDOC can be visualized through the line chart. It provides a meaningful reference index for clinicians to evaluate the prognosis of patients with pDOC.

## 2 Methods

### 2.1 Data collection and clinical evaluation

A total of 134 patients diagnosed with pDOC, sequentially admitted to the Department of Neurosurgery at the Fifth Affiliated Hospital of Zhengzhou University between January 2022 and March 2023, were included in our research. Inclusion criteria: (1) No scalp lesions or intracranial metal implants; (2) No history of neurological diseases or mental disorders; (3) No onset period of acute disease or chronic disease; (4) The patient has lost consciousness for more than 28 days. Exclusion criteria: (1) Presence of intracranial anterior and posterior lobe lesions; (2) History of implantation of pacemaker, aneurysm clip or other metal devices; (3) Large skull defects caused by various causes lead to scalp depression which significantly affects the fit between the scalp and the brain electrode cap. Our collected data included not only the gender, age, etiology (including traumatic, non-traumatic, and anoxic etiologies), CRS-R score, and bloodroutine at admission, but also liver function, coagulation, blood glucose, esting EEG data, and CRS-R scores after patient discharge. The current research followed the guidelines of the Helsinki Declaration and received approval from the ethics committee of Zhengzhou University’s Fifth Affiliated Hospital in China (KY2020024,02/11/2020). Informed consent was obtained from the guardians of all subjects participating in this study and signed informed consent forms.

### 2.2 EEG collection

EEG data were recorded using 32 electrodes according to 10/20 International System (Nicolet EEG V32, Natus, United States). The notch filtering of the signals was performed at 50 Hz, with the recorder’s band-pass filter operating within a range of DC to 1,000 Hz. The EEG signals were converted into digital format at a rate of 2.5 kHz while ensuring that the skin impedance of all electrodes did not exceed 5 kΩ. At the time of the EEG recording, the patients were positioned in their hospital beds with earplugs in, limiting any noise. The EEG recordings were conducted as patients were behaviorally alert and kept their eyes open. Each EEG recording session lasted approximately 10 min.

### 2.3 EEG signal preprocessing

The preprocessing of EEG data mainly includes the following steps: (1) Referring to the previous microstate study (Lehmann and Michel, 2011; Michel and Koenig, 2018), a finite impulse response finiteimpulseresponse (FIR) filter is used to perform 2–20 Hz bandpass filtering on each channel to eliminate the influence of high frequency noise, because the microstate is several different quasi-steady states in the alpha band (8–12 Hz) of resting EEG signals. (2) Independent component analysis (ICA) was used to remove electro-oculogram (EOG). (3) Manual removal of bad channels and tests (physical exercise, muscle activity). (4) The EEG data were re-referenced and converted into common average reference.

### 2.4 Microstates analysis

EEG signals were analyzed offline in the EEGLAB 12.0.2.5b environment using MATLAB 2016b software from MathWorks, based in Natick, Massachusetts, United States. The microstate analysis involved importing preprocessed EEG signals into the Microstate EEGLAB Toolbox for the analysis and calculation of relevant parameters (Guo et al., 2022). By computing the global field potential (GFP) value at every moment in the time series, we could identify the point with the highest signal-to-noise ratio (SNR). The calculation formula is as follows:


$$GFP=\frac{\sqrt{\sum_{i}^{k}{\left[Vi\left(t\right)-V_{\mathrm{mean}}\left(t\right)\right]}^{2}}}{k}$$

The GFP curve is a tool for determining the brain’s comprehensive reaction to a specific event or swift alterations in brain functionality. The peak of the GFP curve denotes the point at which the field strength is at its optimum and the signal-to-noise ratio in topography is the highest. In resting-state microstate analysis, we found that the map corresponding to the GFP peak is similar to the map of the surrounding time. The similarity between the GFP trough and the surrounding maps is relatively low, suggesting that the transition from one map to another may occur during the negative GFP peak period. Consideration is given to the electric field topology of the GFP curve’s local maximum as a discrete state, and the signal’s evolution is viewed as the ongoing fluctuation and alternation in these states. Therefore, we selected the voltage amplitude at the GFP peak time point for cluster analysis. In this study, we used an improved K-means clustering algorithm for clustering. Microstate analysis includes the following three steps: First, the GFP at each time point is calculated. Then, at the maximum point of each GFP, we can consider the spatial model of the EEG to be stable and occupy most of the time series. Then, an improved K-means clustering method is used to cluster GFP. After microstate analysis, various statistical characteristics of the microstates can be calculated. All time frames of the same microstate class consist of GFP; each microstate class state class coverage, or Cov; and spatial correlation, known as SpatCorr. Other included elements are the frequency of occurrences of each microstate class per second; the mean duration, expressed as MMD; and the global explained variance, otherwise known as GEV. We selected one microstate parameter: frequency of occurrence microstate. However, GFP, MMD, GEV, Cov, and SpatCorr did not show valuable statistical results and were abandoned (see Supplementary material).

### 2.5 Prognosis

The process of regaining consciousness was tracked for a period of 6 months in accordance with patients’ clinical records. This tracking was carried out through phone check-ins and reviewing past instances of hospitalization, as well as examining all related medical records, including those from outpatient follow-up exams. The judgment of prognosis was mainly based on the patient’s CRS-R score (Wannez et al., 2017), which was compared with the patient’s state of consciousness 6 months ago. We classified the main results as good prognosis and poor prognosis. A good prognosis was defined as VS/UWS patients improved to MCS or above at the end of the study, MCS patients improved to EMCS or new changes in consciousness and behavior. If the clinical diagnosis does not improve or even worsen, we classify the clinical results as poor prognosis.

### 2.6 Statistical analysis

Continuous variables are represented in the form of mean ± standard deviation, variables with non-normal distribution are articulated using quartiles, while counts and/or frequencies are used to express classified variables. Continuous variables were subjected to the Shapiro–Wilk test for normality. We utilized univariate logistic regression analysis to sift through the variables relating to clinically significant outcomes. With the significant indices distinguished in the univariate analysis, multivariate logistic regression analysis was then employed for a more in-depth examination. A *p*-value less than 0.05 in the multivariate analysis identifies the variables that independently predict prognosis. We then integrated these four independent variables into the line chart model. The subject working curve’s (ROC) area under the curve (AUC) and the calibration curve in both the training and verification set were utilized. The line chart model’s clinical feasibility was assessed using decision curve analysis (DCA). All research was carried out using R statistical packages (https://www.R-project.org, The R Foundation) and Empower(R) (https://www.empowerstats.com; X&Y Solutions, Inc.). A *p*-value less than 0.05 was considered statistically significant.

## 3 Results

**Figure 1:**
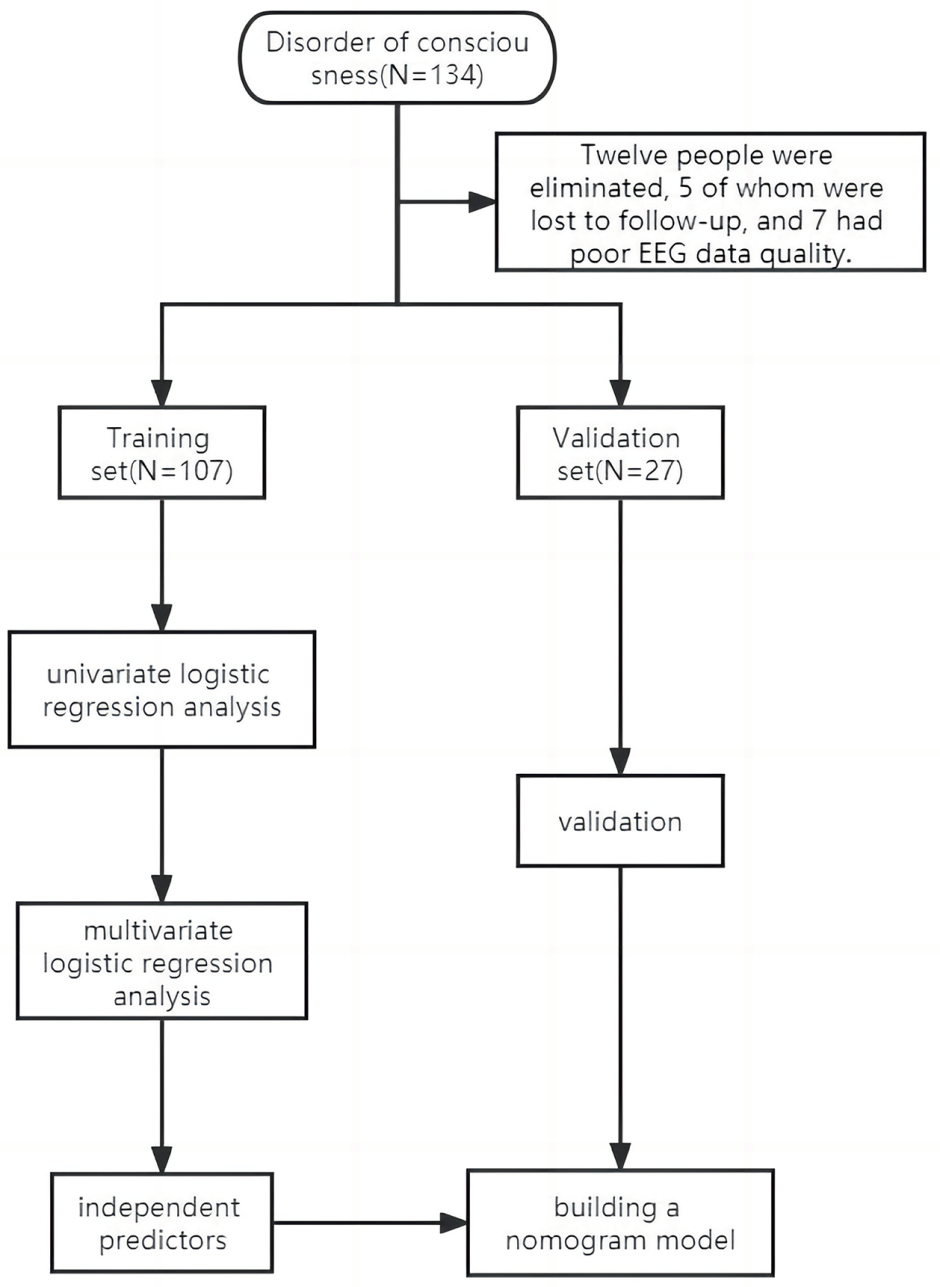
Study flowchart.

The study excluded 12 patients, of whom 5 were lost to follow-up and 7 had poor EEG data. The study comprised 134 patients, and they were divided into two distinct groups: 107 patients formed the training set, treated between January and December 2022, while the validation set included 27 patients treated from January to March 2023. The research object and flow chart are shown in Figure 1.

### 3.1 Basic clinical features of both the training set and the verification set

Table 1 presents the basic clinical characteristics of the training and validation sets, consisting of a total of 134 patients. The mean age was 50 (40–61). No significant differences were observed in the training and validation sets (*p* > 0.05).

**Table 1:** Fundamental clinical features of the training and verification sets.

	Total	Training set	Validation set	*p*-value
Patients (*n* %)	134	107 (79.9)	27 (20.1)	–
Gender (male *n* %)	86	68 (79.1)	18 (20.9)	0.76
Age (years)	50 (40–61)	53 (40–61)	46 (38–55)	0.19
Cause of coma (trauma *n* %)	60	50 (83.3)	10 (16.6)	0.78
State of consciousness at admission (VS/MCS *n* %)	69/65	53 (76.8)/54 (83.1)	16 (23.2)/11 (16.9)	0.83
Diabetes (*n* %)	13	13 (100)	0 (0)	0.06
Hypertension (*n* %)	52	42 (80.8)	10 (19.2)	0.83
Coronary heart disease (*n* %)	11	10 (90.9)	1 (9.1)	0.34
Combined with epilepsy (*n* %)	48	38 (79.2)	10 (20.8)	0.88
Combined with PSH (*n* %)	56	44 (78.6)	12 (21.4)	0.75
Tracheotomy (*n* %)	114	92 (80.7)	22 (19.3)	0.56
Hydrocephalus (*n* %)	58	47 (81.0)	11 (19.0)	0.77
Lymphocyte (10^9^ L)	1.39 (1.01–2.02)	1.47 (1.09–2.01)	1.33 (0.89–2.07)	0.36
Hemoglobin (g/L)	114.37 ± 17.33	114.29 ± 17.26	114.69 ± 17.92	0.92
Glucose (mmol/L)	5.17 (4.56–6.07)	5.25 (4.61–6.21)	5.05 (4.50–5.40)	0.19
Fibrinogen (g/L)	4.46 (3.99–5.00)	4.49 (4.01–5.00)	4.25 (3.88–5.66)	0.77
Albumen (g/L)	33.90 (31.18–37.30)	33.8 (31.10–37.10)	34.4 (32.10–40.10)	0.26
Frequency of Occurrence Microstate A	1.97 (1.17–2.80)	1.96 (1.13–2.80)	2.06 (1.27–2.85)	0.77
Frequency of Occurrence Microstate B	2.14 (0.73–3.17)	2.15 (0.71–3.18)	2.14 (0.98–2.99)	0.98
Frequency of Occurrence Microstate C	2.37 (1.23–3.32)	2.37 (1.29–3.32)	2.37 (0.98–3.33)	0.78
Frequency of Occurrence Microstate D	1.95 (1.02–3.04)	1.98 (1.08–3.121)	1.79 (0.98–2.74)	0.25
CRS-R scale	7 (5–10)	7 (5–10)	6 (4–10)	0.67

### 3.2 Screening factors affecting prognosis

In terms of prognosis results, 54 patients in the training set and 18 patients in the validation set had a poor prognosis after 6 months of follow-up. Univariate analysis showed that fibrinogen level, CRS-R score, frequency of occurrence microstate A, and paroxysmal sympathetic hyperactivity (PSH) attacks were associated with the prognosis of pDOC (Table 2).

**Table 2:** Univariate analysis of prognosis.

	Total	Favorable prognosis	Unfavorable prognosis	*p*-value
Patients (*n* %)	107	53 (49.5)	54 (50.5)	
Gender (male *n* %)	68	35 (51.4)	33 (48.5)	0.597
Age (years)	50.22 ± 14.23	51.45 ± 12.01	49.02 ± 16.14	0.378
Cause of coma (trauma *n* %)	50	24 (48.0)	26 (52.0)	0.551
Diabetes (*n* %)	13	5 (38.4)	8 (61.5)	0.36
Hypertension (*n* %)	42	24 (57.1)	18 (42.9)	0.21
Coronary heart disease (*n* %)	10	6 (60.0)	4 (40.0)	0.49
Combined with epilepsy (*n* %)	38	16 (42.1)	22 (57.9)	0.25
Combined with PSH (*n* %)	44	12 (27.3)	32 (72.7)	0.01
Tracheotomy (*n* %)	92	47 (48.9)	47 (51.1)	0.75
Hydrocephalus (*n* %)	47	22 (46.8)	25 (53.2)	0.62
Lymphocyte (10^9^ L)	1.47 (1.09–2.01)	1.43 (1.05–2.18)	1.48 (1.15–1.87)	0.99
Hemoglobin (g/L)	114.29 ± 17.26	113.21 ± 15.46	115.35 ± 18.94	0.525
Glucose (mmol/L)	5.25 (4.61–6.21)	5.28 (4.71–6.27)	5.09 (4.39–6.20)	0.39
Fibrinogen (g/L)	4.49 (4.01–5.00)	4.90 (4.29–5.43)	4.12 (3.87–4.66)	0.01
Albumen (g/L)	33.72 ± 4.79	33.64 ± 4.39	33.81 ± 5.20	0.854
Frequency of Occurrence Microstate A	1.96 (1.13–2.79)	1.73 (0.77–2.27)	2.38 (1.58–3.06)	0.001
Frequency of Occurrence Microstate B	2.15 (0.71–3.18)	2.04 (0.74–3.22)	2.16 (0.71–3.16)	0.876
Frequency of Occurrence Microstate C	2.37 (1.29–3.32)	2.00 (1.22–3.48)	2.62 (1.59–3.15)	0.774
Frequency of Occurrence Microstate D	1.98 (1.08–3.12)	1.87 (0.76–3.10)	2.20 (1.60–3.19)	0.239
CRS-R scale	7 (5–10)	9 (6–11)	5 (4–8)	0.01

**Figure 2:**
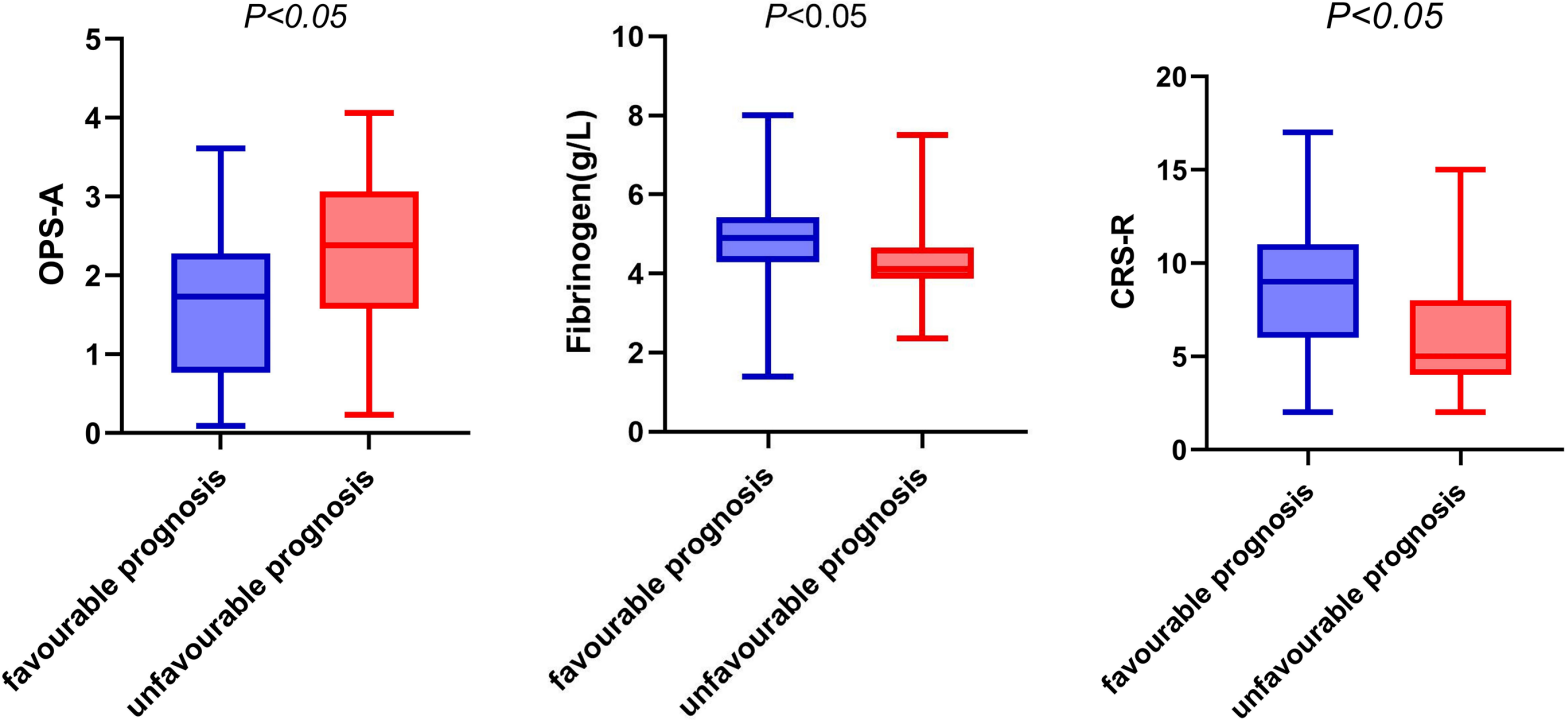
Box diagram of fibrinogen, OPS-A, CRS-R and prognosis. OPS-A, frequency of occurrence microstate A.

In the group with a poor prognosis, the fibrinogen level, CRS-R score, and frequency of occurrence microstate A were all different compared to the good prognosis group. Specifically, the fibrinogen level and CRS-R score were lower, while the frequency of occurrence microstate A was higher in the poor prognosis group (refer to Figure 2).

Multivariate logistic regression analysis showed that fibrinogen level, CRS-R score, frequency of occurrence microstate A, and PSH attack were independent prognostic factors of pDOC (Table 3).

**Table 3:** Multivariate regression analysis.

	*β*	S.E.	Wald	*p*-value	OR	95%C.I.	
						Lower	Upper
Frequency of Occurrence Microstate A	0.837	0.282	8.793	0.003	2.31	1.328	4.016
PSH	−1.574	0.517	9.278	0.002	0.207	0.075	0.571
CRS-R	−0.256	0.088	8.462	0.004	0.774	0.652	0.92
Fibrinogen (g/L)	−0.73	0.236	9.616	0.002	0.482	0.304	0.764

### 3.3 Development and validation of the model

**Figure 3:**
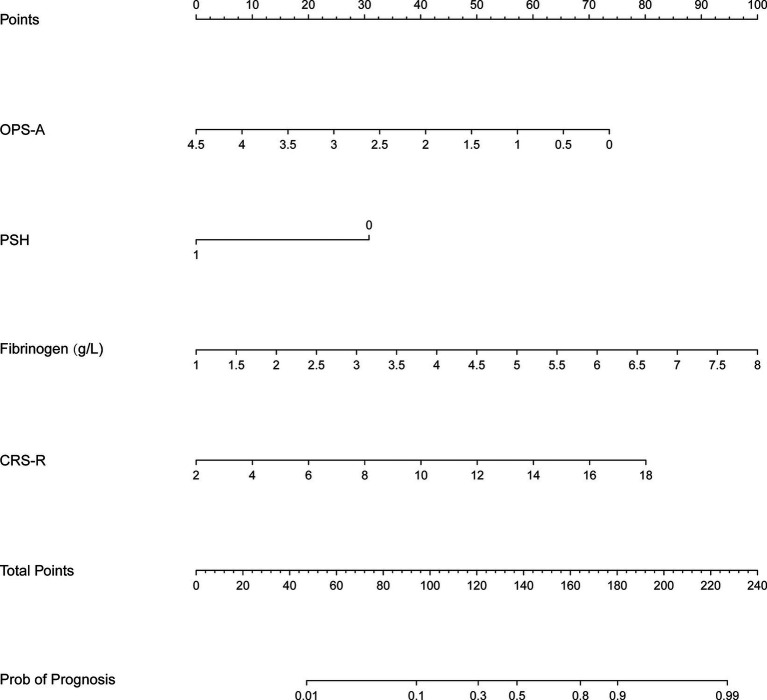
Prediction nomogram model. OPS-A, frequency of occurrence microstate A.

According to the results of the logical regression analysis, four predictive variables (fibrinogen level, CRS-R score, frequency of occurrence microstate A, and PSH) and one outcome variable (unfavorable prognosis) were used to construct a 6 month prediction nomogram model for patients with pDOC (Figure 3). The sum of scores for each prediction variable needs to be determined. As the total score increases, so does the likelihood of negative outcomes.

### 3.4 Calibration and decision curve

**Figure 4:**
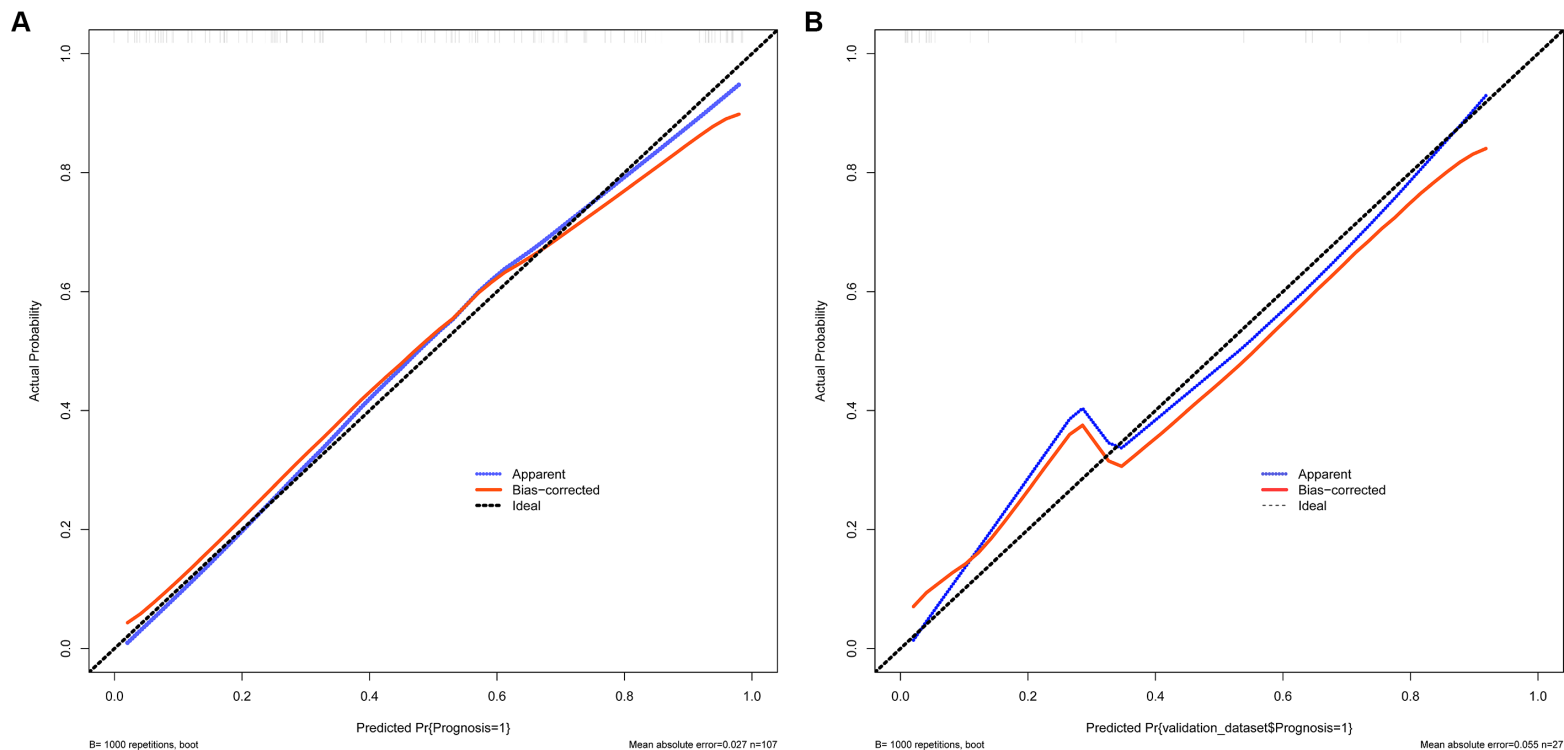
**(A,B)** Show the calibration curves used to measure calibration. These curves demonstrate that the actual risk aligns with the predicted risk in both the training and validation sets. **(A)** Training set and **(B)** Validation set.

The calibration curve is employed as a measurement for calibration to evaluate the correlation between the real risk and forecasted risk, which is a key feature of predictive models. The results show that the actual risk (red line) and predicted risk (blue line) are consistent in both the training and validation cohorts (Figures 4A,B).

**Figure 5:**
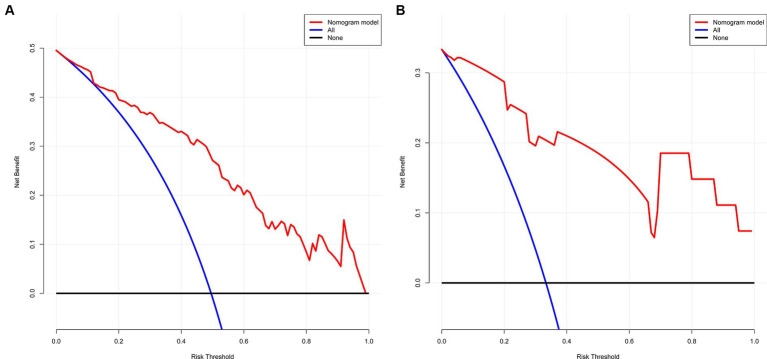
**(A,B)** The net gain of the model surpasses the net gain of all treatments at an equivalent threshold probability within both training and validation sets, as shown in decision curve. **(A)** Training set and **(B)** Validation set.

The decision curve shows that the model’s net benefit (indicated by the red curve) surpasses the net benefit from all treatments at the same threshold probability in both the training and validation sets (as shown by the blue curve) (see Figures 5A,B). This suggests that this model could be beneficial for clinicians in their decision-making process.

### 3.5 ROC curve to evaluate the discriminative ability

**Figure 6:**
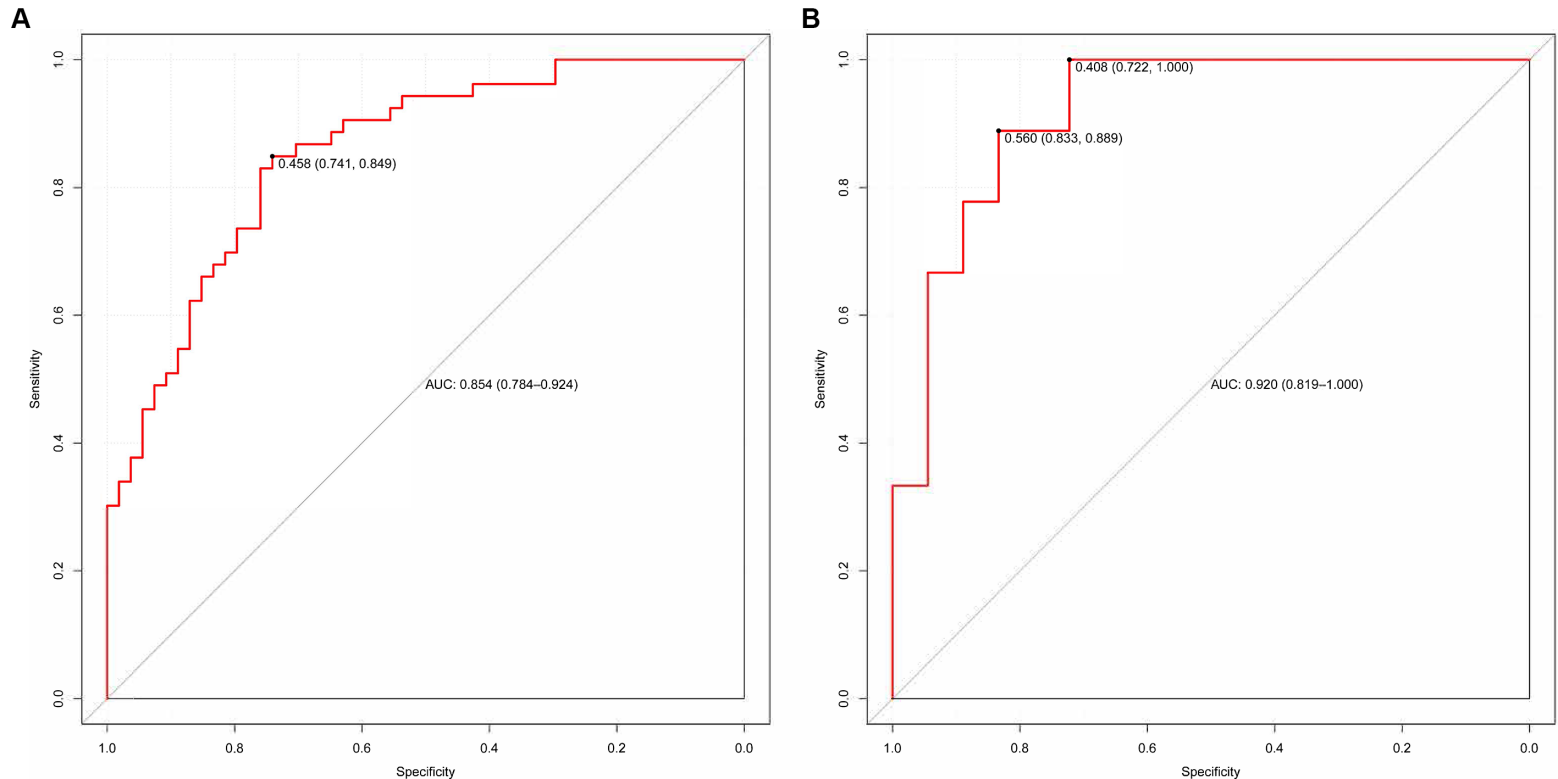
**(A,B)** The ROC curve used to assess the discriminative ability is displayed. **(A)** Training set and **(B)** Validation set.

We evaluated the predictive model across the training and validation sets. The power of discrimination in the model pertains to its aptitude to accurately differentiate non-events from events, assessed using the area under the curve (AUC) metric. As depicted in Figures 6A,B, the computed AUC score of the nomogram for the training group was found to be 0.854, with a 95% confidence interval (CI) of 0.784 to 0.924. For the validation group, the AUC score was observed to be 0.920, with a 95% CI between 0.819 and 1.000. These results demonstrate that the model has good discriminative ability.

## 4 Discussion

Studies have shown that the prognosis of pDOC should be based on multiple variables to optimize the accuracy (Giacino et al., 2018). Although a large number of previous studies have focused on determining the clinical factors affecting poor prognosis, a model that can predict the poor prognosis of pDOC is still needed. Our results showed that the frequency of microstate A, CRS-R score, PSH attack and fibrinogen level were associated with poor prognosis at 6 months. Based on these four variables, an adverse outcome prediction model based on EEG microstate and clinical parameters of pDOC was established. In addition, we use calibration curves and DCA to evaluate the calibration and clinical benefits of the two dataset models. Our results show that the model is practical for predicting adverse outcomes and assisting clinical decision-making in patients with pDOC.

The prognosis of patients with pDOC is known to be influenced by numerous factors (Formisano et al., 2019). Studies have shown that younger age, MCS diagnosis at admission, higher serum albumin levels, and the presence of pupil reflex are associated with improved prognosis at 6 months after brain injury (Xiong et al., 2022). Several studies have shown that young age, women (Estraneo et al., 2019), cases of traumatic venereal diseases (Whyte et al., 2009), and higher CRS-R (Portaccio et al., 2018) are associated with better clinical results. At present, many studies focus on the influence of clinical factors on the prognosis of pDOC. However, this approach is highly subjective, and there are few studies that combine clinical factors with EEG objective data to explore their influence on the prognosis of pDOC. Therefore, this study comprehensively evaluated the effect of clinical factors combined with EEG objective indicators on the prognosis of pDOC.

EEG is a method used to scrutinize the physiological activity of distinct brain regions. This is accomplished by documenting the potential and intensity of the electric field from electrodes positioned on the scalp’s surface (von Wegner and Laufs, 2018). At present, there are some relatively mature methods to extract effective information from multi-channel EEG data, and microstate analysis is one of them (Britz et al., 2010). Different microstates corresponded to different brain regions and brain network changes. Microstate A is mainly connected with bilateral superior temporal gyrus and middle temporal gyrus, which is related to the components of these two auditory systems, indicating the process of auditory information intake and processing (Britz et al., 2010). Microstate B interacts with a wide range of brain regions associated with visual processing, suggesting that it may be part of the visual network (Khanna et al., 2015). Microstate C is related to the posterior anterior cingulate cortex, bilateral inferior frontal gyrus and right anterior cingulate cortex, and plays an important role in promoting the transition between the central executive network and the default mode network (Khanna et al., 2015). Microstate D is related to the region located in the right prefrontal lobe and inferior parietal lobe and is responsible for high-level tasks such as cognition and decision-making in the central executive network (Khanna et al., 2015). After studying the time dynamics of EEG sources in patients with pDOC for the first time, Zhang et al. (2023) found seven microstates with different spatial distributions of cortical activation. Notable variations were observed in microstates between the MCS group and the VS group. Representative research has shown that microstate metrics can provide a new basis for assessing patients with pDOC and predicting their prognosis. Stefan et al. (2018) in his analysis of EEG in 63 pDOC patients, made prognostic predictions for 39 patients and identified notable discrepancies in microstate A. Similarly, Guo et al. (2022) alongside other researches, discovered that there were significant alterations to microstate C and microstate D in pDOC patients following high-definition transcranial direct current stimulation (HD-tDCS). In our study, we used frequency of occurrence microstate as an indicator to predict the outcome of pDOC. Our discovery showed that a high frequency of occurrence microstate A not only signifies a grim prognosis but also independently contributes to a deteriorating outcome. Our results have similarities with Stefan’s study. Microstate A is mainly related to the intake and processing of auditory information, and the frequency of occurrence of microstates can reflect the activation trend of underlying neural generators, increase may be a signal of neurological dysfunction. To the best of our knowledge, this is the first time microstate analysis has been utilized for result prediction in this manner, highlighting uncharted potential for this method.

At present, the gold standard for evaluating the degree of consciousness impairment, building a platform for differential diagnosis, prognostic evaluation, and formulating a sensible treatment plan for consciousness impairments, is the CRS-R score. Some studies have found that CRS-R score is an independent factor affecting the recovery of consciousness in patients with pDOC, and those with lower scores are less likely to recover consciousness (Lucca et al., 2019). Another study examined long-term survival and functional outcomes in patients with pDOC 1–8 years after brain injury and found that higher CRS-R scores were associated with a favorable prognosis (Liu et al., 2023). The above studies suggest that CRS-R is one of the important indicators for assessing the prognosis of pDOC. Our study revealed that a lower CRS-R score was associated with a poor prognosis. Furthermore, a multifactorial regression analysis demonstrated that a low CRS-R score was an independent risk factor for poor prognosis, which aligns with the findings of this study.

Paroxysmal sympathetic hyperactivity (PSH) is a syndrome typically seen in medical complications that is distinguished by irregular activity in the sympathetic nervous system or motor functions. This condition is known to respond to non-painful stimuli and is frequently observed in individuals with severe traumatic brain injury (Baguley et al., 2014). However, there is limited research available on PSH in patients with impaired consciousness. The current literature states that the occurrence of PSH in TBI patients ranges from 8 to 33% (Jafari et al., 2022). This study reports a slightly higher incidence of 41.8%; however, this is still relatively comparable to prior research. Presently, two theories exist regarding the mechanism of PSH. One postulates that following an injury, the path of the anterior descending branch of the spinal cord, which consists of the cortical inhibition center, hypothalamus, diencephalon, and brainstem, becomes obstructed, thereby suppressing sympathetic activity. The other hypothesis proposes that the inhibitory–excitatory loop of the spinal cord is uninhibited, leading to an increase in the output of sympathetic activity and resulting in PSH. Secondly, PSH may be associated with neuroendocrine disorders. When the secretion of adrenocortical hormone and corticotropin decreases, the increased secretion of release factor may result in a heightened adrenaline stress response (Zheng et al., 2020). PSH is a risk factor for deterioration in neurological outcomes in patients with TBI (Lucca et al., 2021). The presence of PSH has a connection to the clinical outcome for patients dealing with persistent consciousness impairments. Choi et al. (2013) suggests that the main risk factor for the occurrence of PSH is the severity of brain injury. Perkes et al. (2010) has pointed out that most patients with PSH have a GCS score of less than 8, or even as low as 4, indicating that the development of PSH is more likely with more severe brain injuries. Because the brain injury in patients with PSH is usually more severe, it may affect the recovery of consciousness. Caplan et al. (2015) also found that patients with PSH had a higher incidence of persistent vegetative state and took longer to regain consciousness after coma. Our study found that patients with PSH attacks had a poor prognosis. In this study, through univariate and multivariate analysis, it was found that PSH was a risk factor for adverse effects, which was consistent with the results of previous studies.

Clinical laboratory examination is also an important part of the evaluation of disturbance of consciousness. It can reflect the general condition of patients, including the necessary blood coagulation function test. The progression of intracranial hemorrhage and the negative outcomes post TBI are significantly influenced by dysfunction in coagulation (Nakae et al., 2022a). Among the different elements impacting the blood coagulation process, fibrinogen is of utmost importance as it promotes platelet aggregation and acts as the main substrate for plasma coagulation. It forms a reticular network that enhances the strength of blood clots (Sorensen et al., 2012). Hypofibrinogenemia, characterized by low levels of fibrinogen (below 2.29 g/L), is frequently observed in trauma patients, with 50–74% presenting with this condition upon admission. This condition is strongly linked to increased mortality rates (Hagemo et al., 2014). In trauma patients, hypofibrinogenemia serves as a significant sign of a negative outcome (Nakae et al., 2022b). Studies have found that 38.6% of TBI patients have a fibrinogen concentration < 2 g/L on admission, which is closely related to an increase in in-hospital mortality (Lv et al., 2020). In TBI models, damaged axons are an important source of fibrinogen leakage. A local increase in fibrinogen levels causes microglia to gather around damaged blood vessels, which in turn promotes a continuous acute microglial response and induces microglia to release reactive oxygen species, resulting in axonal injury (Merlini et al., 2019). This research determined that diminished fibrinogen levels negatively impacted the prognosis for patients experiencing pDOC. The fibrinogen levels measured 4.90 g/L for patients with a favorable prognosis and 4.12 g/L for those with an unfavorable prognosis, both exceeding the 2 g/L rate. Despite constituting an unfavorable prognostic indicator, this study’s value was somewhat higher compared to other similar research. The explanation for this could be that the individuals participating in this study are patients suffering from persistent consciousness disturbances and non-acute brain injuries. Additionally, the course of the disease in these patients is longer than 28 days, which means that this value could be influenced by many factors.

Our model utilizes readily available variables that can be readily applied in clinical practice. For the first time, we evaluated the prognostic value of patients with pDOC from EEG microstate combined with clinical behavior, complications, and erological markers. The line chart illustrates its outstanding predictive performance in both the training and verification groups. The AUC values were 0.854 for the training cohort and 0.920 for the verification cohort, indicating superior predictive capability. In addition, we used calibration curves and DCA to evaluate the calibration and clinical effectiveness of the model in both datasets. Our results show that the model is cost-effective in predicting the prognosis of patients with pDOC and assisting clinical decision-making.

There are certain limitations to our research. Firstly, the small sample size may have resulted in an underestimation of the value of certain predictors. Furthermore, the brief duration of the follow-up period was insufficient to pinpoint long-term prognostic indicators. This is because it has been found that prognostic markers may have different effects on each marker. Additionally, this study was a single-center study, lacking external verification. Therefore, there is a need for multi-center external verification to better demonstrate the prediction accuracy of the model.

## 5 Conclusion

A higher frequency of occurrence microstate A, a lower level of consciousness, a lower level of fibrinogen and the occurrence of PSH can predict a poor prognosis of pDOC at 6 months. EEG microstate analysis combined with clinical factors to establish an adverse prognosis prediction model for patients with pDOC provides a new objective and reliable tool for predicting the prognosis of pDOC, has significant practical value in clinical decision-making, and is helpful for clinicians to provide personalized treatment decisions for pDOC patients and their families.

## Data availability statement

The original contributions presented in the study are included in the article/Supplementary material, further inquiries can be directed to the corresponding author.

## Ethics statement

The studies involving humans were approved by the Ethics Committee of Zhengzhou University’s Fifth Affiliated Hospital in China. The studies were conducted in accordance with the local legislation and institutional requirements. The participants provided their written informed consent to participate in this study. Written informed consent was obtained from the individual(s) for the publication of any potentially identifiable images or data included in this article.

## Author contributions

WL: Writing – review & editing, Writing – original draft, Methodology. YG: Writing – review & editing, Writing – original draft. JX: Writing – review & editing, Data curation. YW: Writing – review & editing, Formal analysis, Data curation. DZ: Writing – review & editing, Validation, Methodology, Investigation. ZX: Writing – review & editing, Validation, Software, Methodology, Formal analysis. XF: Writing – review & editing, Supervision. SZ: Writing – review & editing, Software, Investigation, Conceptualization. HZ: Writing – review & editing, Formal analysis, Data curation. XW: Writing – review & editing, Supervision, Resources, Funding acquisition, Conceptualization.
