# Supplementary material for: Establishment and validation of a bad outcomes prediction model based on EEG and clinical parameters in prolonged disorder of consciousness
Source: Front Hum Neurosci. 2024 Jun 17;18:1387471. doi: 10.3389/fnhum.2024.1387471 (PMC11215084; doi:10.3389/fnhum.2024.1387471)
Supplement: Supplementary file 1 [file Table_1.DOCX]

**Attachment:**

**Table: Analysis of EEG microstate and prognosis**

|  | Class A | | | Class B | | | Class C | | | Class D | | |
| --- | --- | --- | --- | --- | --- | --- | --- | --- | --- | --- | --- | --- |
|  | Favourable prognosis | Unfavourable prognosis | *P*-value | Favourable prognosis | Unfavourable prognosis | *P*-value | Favourable prognosis | Unfavourable prognosis | *P*-value | Favourable prognosis | Unfavourable prognosis | *P*-value |
| Mean GFP(mV) | 8.63 (6.3-11.13) | 7.57 (5.08-10.47) | 0.29 | 7.16 (5.41-14.59) | 6.67 (4.15-9.58) | 0.054 | 8.36 (5.45-11.57） | 8.26 （6.00-11.48） | 0.87 | 7.57 （4.50-11.18) | 7.54 (5.22-9.31) | 0.61 |
| MMD(s) | 76.07 (61.54-120.29） | 93.29 (67.28-128.69) | 0.27 | 97.33 (64.91-135.91) | 84.79 (61.81-124.99) | 0.27 | 96.89 (74.32-128.95) | 92.91 (71.37-117.84) | 0.5 | 88.94 (63.50-122.49) | 99.89 (71.33-154.31) | 0.33 |
| Coverage(%) | 0.13 (0.04-0.30) | 0.24 (0.10-0.39） | 0.09 | 0.27 （0.04-0.38） | 0.20 （0.03-0.35） | 0.19 | 0.23 （0.10-0.47） | 0.21 （0.09-0.36） | 0.28 | 0.25 (0.04-0.41) | 0.28 (0.10-0.43） | 0.54 |
| GEV | 0.05 （0.004-0.19） | 0.05 （0.01-0.22） | 0.51 | 0.08 （0.004-0.19） | 0.04 （0.002-0.11） | 0.13 | 0.06 （0.02-0.18） | 0.05 （0.01-0.14) | 0.26 | 0.07 (0.006-0.17) | 0.06 （0.008-0.18） | 0.75 |
| SpataCorr | 0.50  (0.33-0.68) | 0.50  (0.33-0.64) | 0.71 | 0.57  (0.37-0.64) | 0.47  (0.30-0.62) | 0.18 | 0.56  (0.38-0.61) | 0.48  (0.32-0.59) | 0.10 | 0.53  (0.37-0.59) | 0.49  (0.34-0.61) | 0.92 |
